# Supplementary material for: G-protein βγ subunits determine grain size through interaction with MADS-domain transcription factors in rice
Source: Nat Commun. 2018 Feb 27;9:852. doi: 10.1038/s41467-018-03047-9 (PMC5829230; doi:10.1038/s41467-018-03047-9)
Supplement: Supplementary file 1 — Supplementary Information [file 41467_2018_3047_MOESM1_ESM.pdf]

**Supplementary Information**

G-protein βγ subunits determine grain size through interaction with MADS-domain transcription factors in rice

Liu et al.

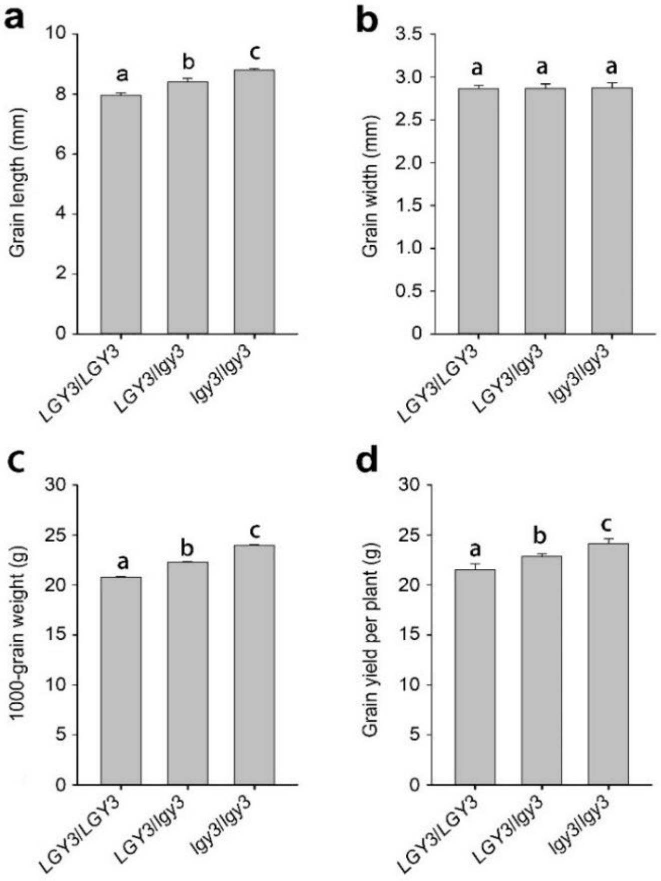


**Supplementary Figure 1. The semi-dominant *qlgy3* allele from the *japonica* variety L-204 was associated with the formation of long-grain and high yield.** (**a**) Grain length. (**b**) Grain width. (**c**) 1,000-grain weight. (**d**) Grain yield per plant. All phenotypic data were measured from the paddy-grown $BC_4F_2$ progenies derived from the cross between RIL186 and HJX74 (the recurrent parent) under normal cultivation conditions. Segregation of the *lgy3/lgy3:* homozygotes for the L-204 *qlgy3* allele; *LGY3/lgy3*: heterozygotes for the L-204 *qlgy3* allele and the HJX74 *qLGY3* allele; *LGY3/LGY3*: homozygotes for the HJX74 *qLGY3* allele. Data shown as mean ± s.e.m. (n = 288). Statistical analyses were performed by Duncan's multiple range tests, the same lowercase letter denotes a non-significant difference between the means ($P$ > 0.05).

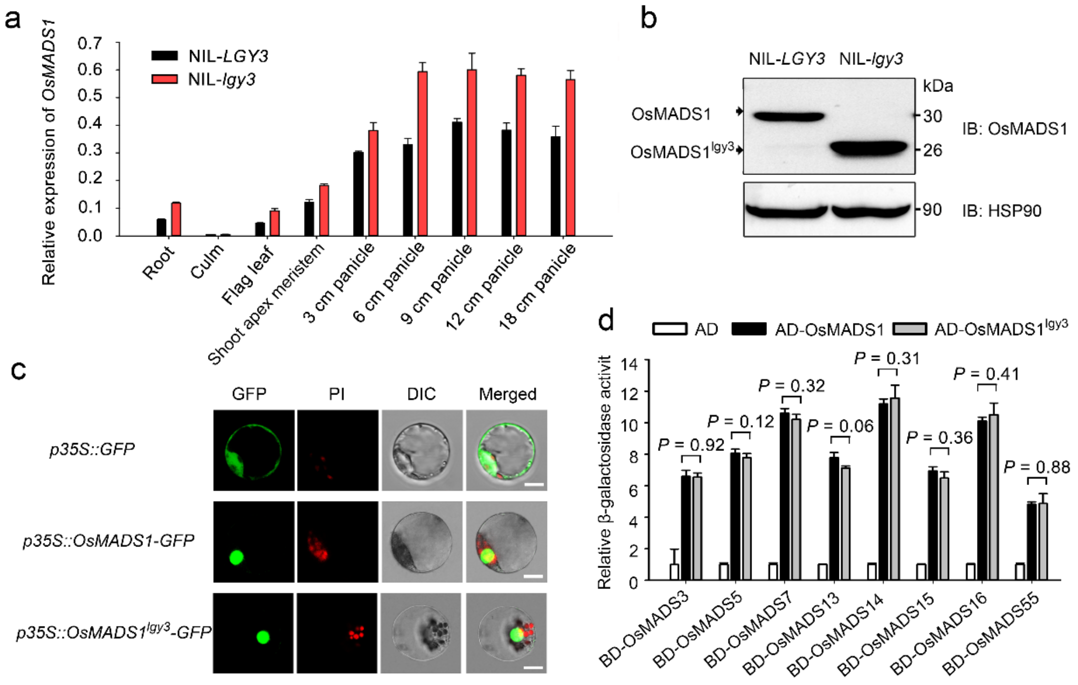


**Supplementary Figure 2. The effects of the truncating splice site mutation on gene expression and function of *OsMADS1*.** (**a**) Expression patterns of *OsMADS1* in different organs of the NIL plants in the *indica* rice variety RD23. Relative expression levels were expressed as the relative copies of rice *Actin3*. Data shown as mean ± s.e.m. (n = 3). (**b**) Immunoblot of OsMADS1. The total proteins were extracted from young panicles (3 to 6 cm length). The abundance of HSP90 protein detected by anti-HSP90 antibodies was used as loading control. Molecular weight markers are indicated in kDa. (**c**) Localization of OsMADS1-GFP and OsMADS1$^{lgy3}$-GFP fusion proteins. Scale bar, 10 μm. (**d**) Yeast two-hybrid assays. The empty vector *pGADT7* was used as negative control. Data shown as mean ± s.e.m. (n = 3). A Student's *t*-test was used to generate the *P* values.

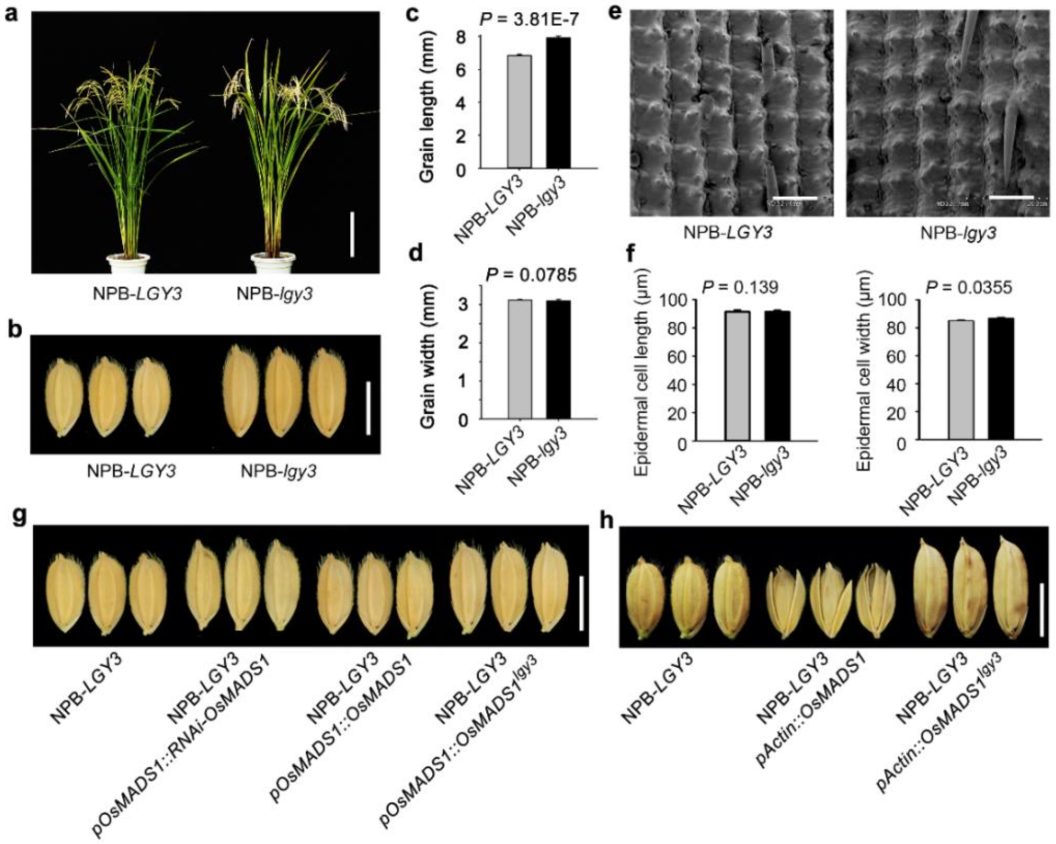


**Supplementary Figure 3. Natural variations of *OsMADS1* regulated grain size**. (**a**) The gross morphology of the NIL plants. Scale bar: 20 cm. (**b**) Grain size and shape. Scale bar: 5 mm. (**c**) Grain length. Data shown as mean ± s.e.m. (n = 30). (**d**) Grain width. Data shown as mean ±s.e.m. (n = 30). (**e**) Scanning electron microscope analysis. Scale bar: 100 µm. (**f**) Average length and width of outer epidermal cells shown in **e**. Data shown as mean ±s.e.m. (n = 30). (**g** and **h)**, Grain size and shape. Scale bar: 5 mm. A Student's *t*-test was used to generate the *P* values (Panels **c**, **d** and **f**).

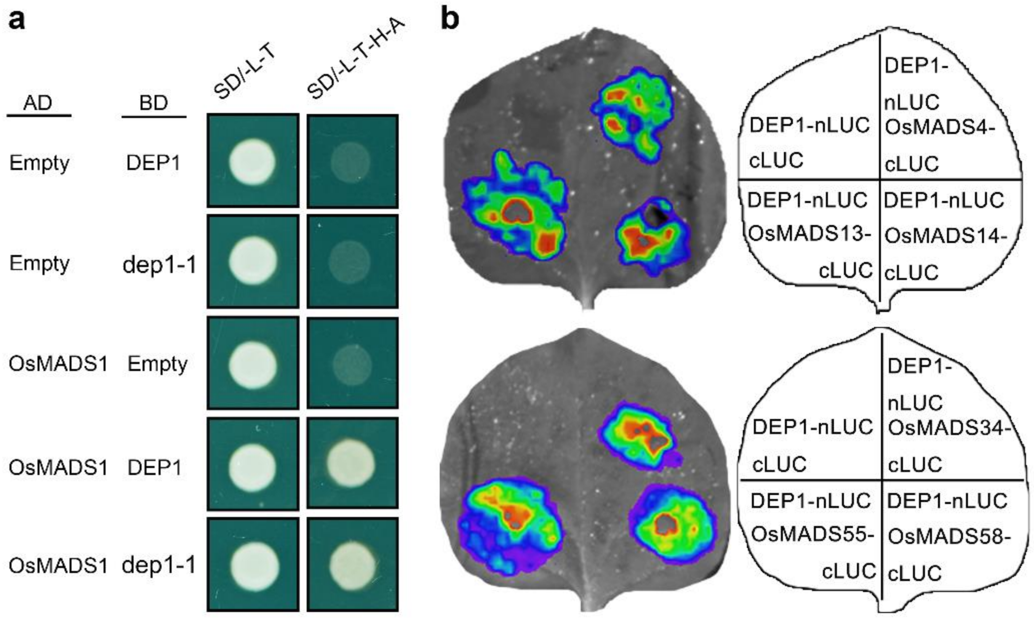


**Supplementary Figure 4. The DEP1 protein interacted directly with a number of MADS-domain transcription factors.** (**a**) Yeast two-hybrid assays. The transformant co-transformed with empty vectors pGADT7 (AD), and pGBDT7 (BD) was used as negative control. (**b**) The split firefly luciferase complementation assays. The cLUC-tagged OsMADS4, OsMADS13, OsMADS14, OsMADS34, OsMADS55, or OsMADS58, was co-transformed into tobacco leaves along with the nLUC-targeted DEP1.

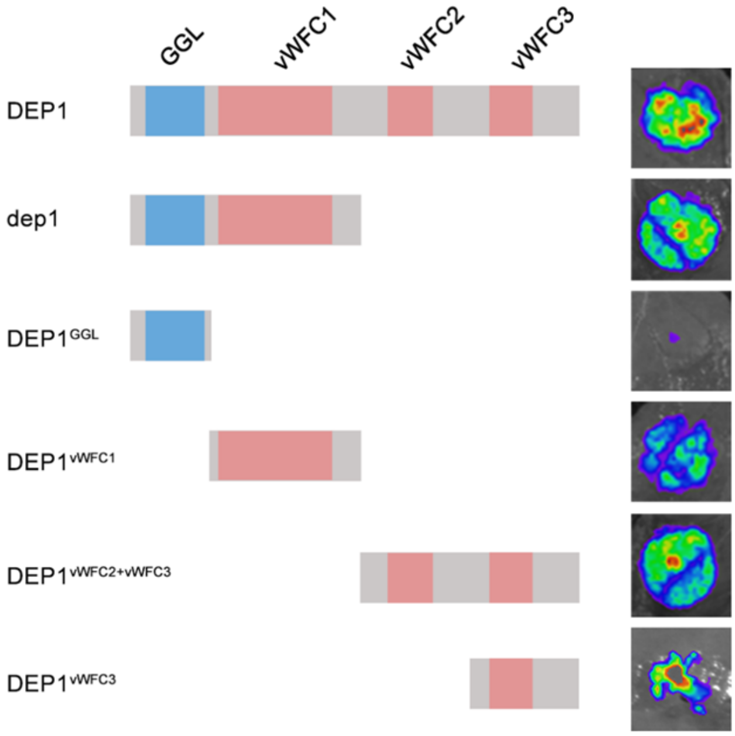


**Supplementary Figure 5. The vWFC domain of DEP1 is necessary and sufficient for the interaction with OsMADS1.** The image shows the split firefly luciferase complementation assays, in which nLUC-tagged deleted and non-deleted versions of DEP1 were co-transformed into tobacco leaves along with cLUC-OsMADS1.

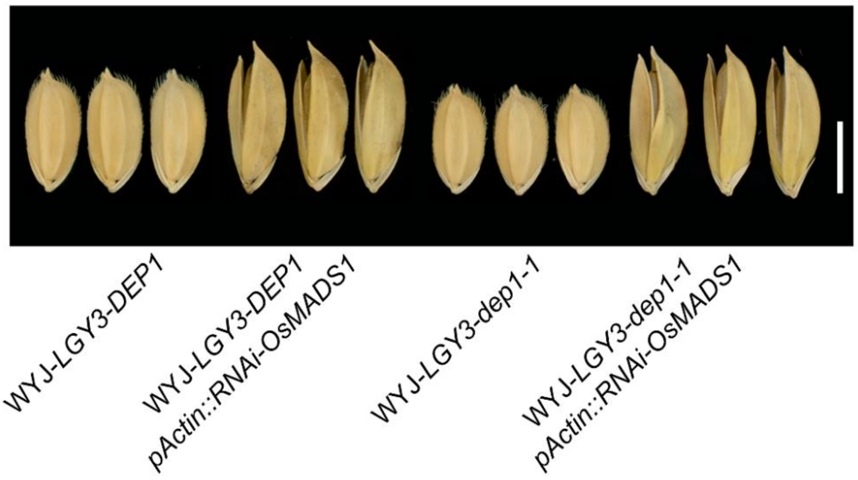


**Supplementary Figure 6. The effect of the constitutive knockdown of *OsMADS1* on grain size and shape in both WYJ7-*LGY3-DEP1* and WYJ-*LGY3-dep1-1* plants.** The transgenic rice plants that had been RNAi-silenced for *OsMADS1* under control of the rice *Actin* promoter exhibited an open flower with the elongated leafy palea and lemma. Scale bar: 5 mm.

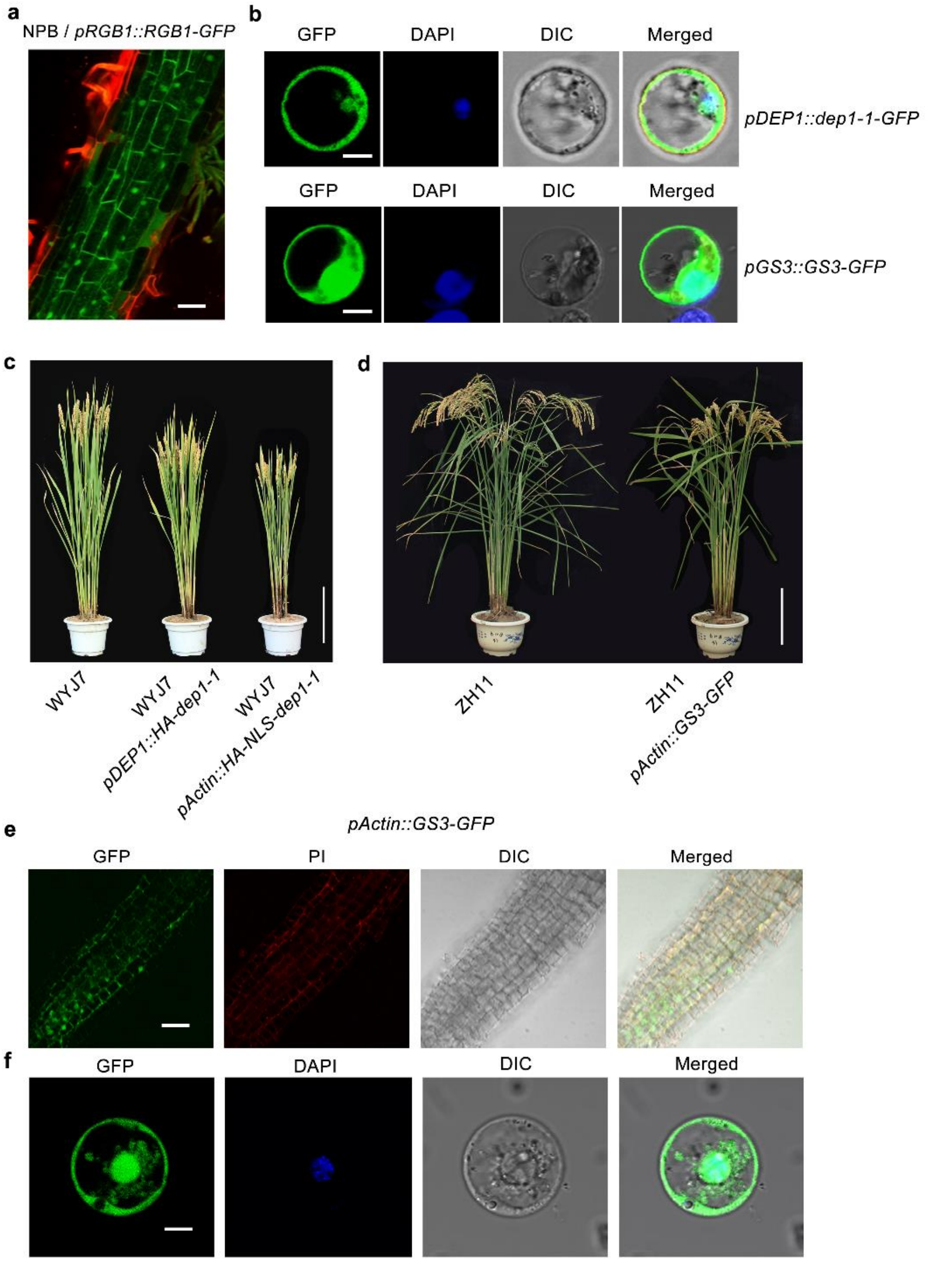


**Supplementary Figure 7. The expression patterns of GFP fusion proteins in the transgenic rice plants.** (**a**) Localization of the RGB1-GFP fusion protein in transgenic Nipponbare plants carrying the *pRGB1::RGB1-GFP* construct. Scale bar: 50 μm. (**b**) The transient expression assays in rice seedlings protoplasts. Scale bar: 15 μm. (**c-d**) Appearance of mature plants. Scale bar: 20 cm. (**e**) The GS3-GFP fusion protein was detectable in the rice root cells. Scale bar: 100 μm. (**f**) The GS3-GFP fusion protein in protoplasts isolated form leaf sheath of 10-day-old seedlings of the transgenic rice plants shown in **e**. Scale bar: 10 μm.

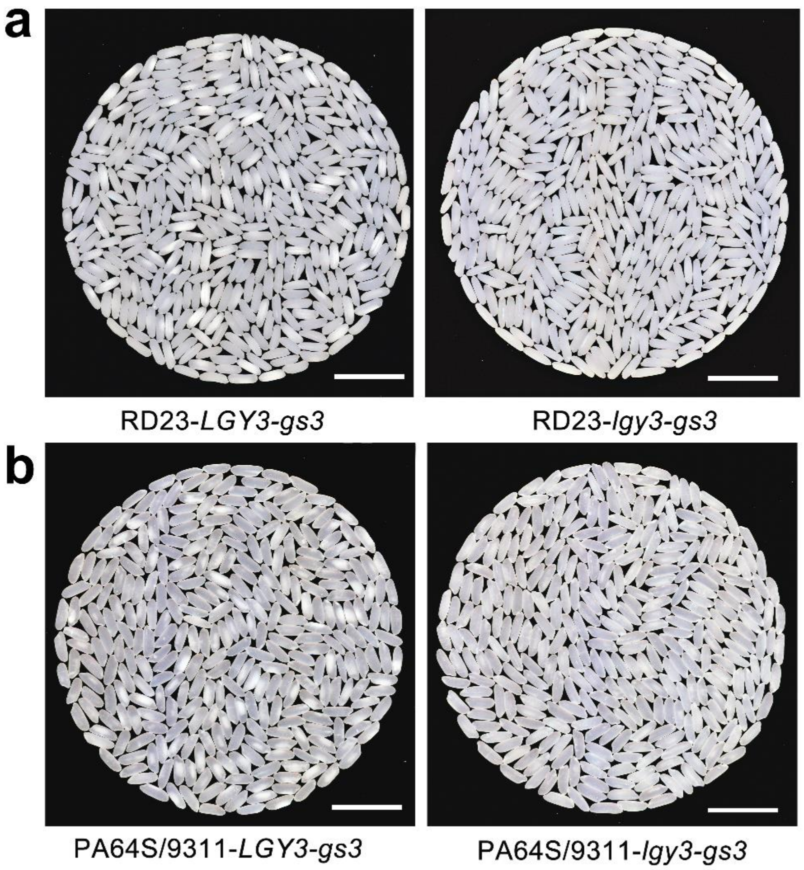


**Supplementary Figure 8. Grain chalkiness and endosperm transparency of the NILs plants. (a**) Grains formed by the RD23-*LGY3-gs3* and RD23-*lgy3-gs3* plants. (**b**) Grain chalkiness and endosperm transparency of the two hybrid combinations (PA64S/9311-*LGY3-gs3* and PA64S/9311-*lgy3-gs3*). PA64S, a photo-thermosensitive genic male sterile line Peiai64S. Scale bar: 15 mm.

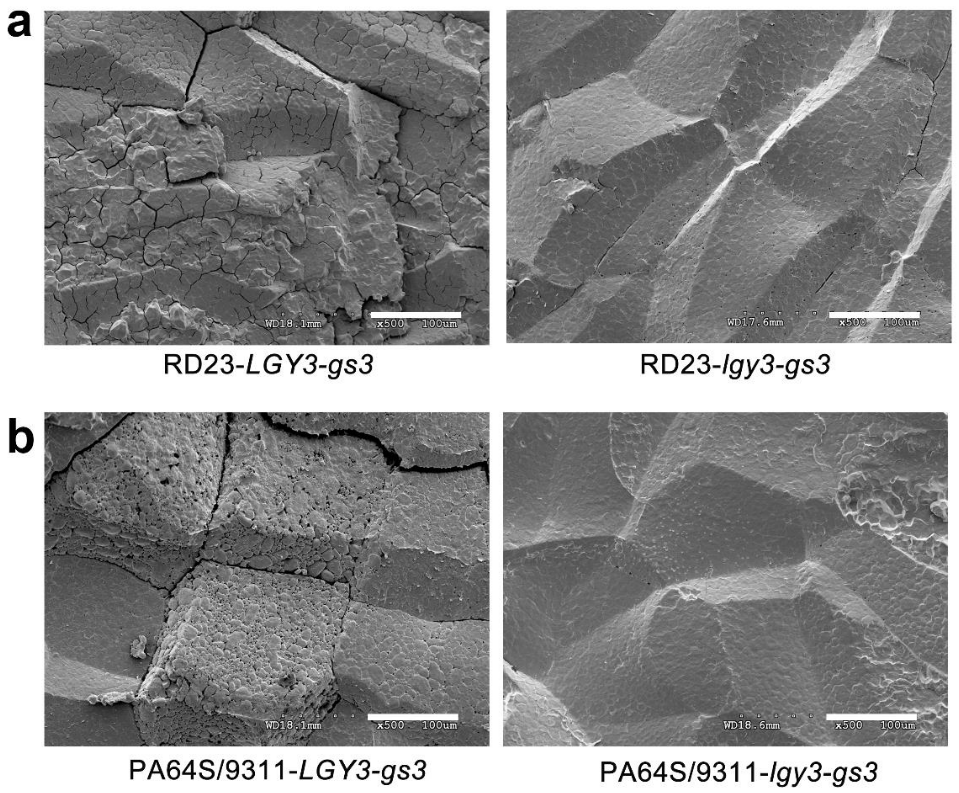


**Supplementary Figure 9. Scanning electron microscopy images of the transverse sections of starch granule from the NILs plants.** (**a**) RD23-*LGY3-gs3* and RD23-*lgy3-gs3* grains. (**b**) PA64S/9311-*LGY3-gs3* and PA64S/9311-*lgy3-gs3* grains. The endosperm of the NILs plants carrying the *lgy3* allele comprised largely sharp edged, compactly arranged polygonal starch granules. PA64S, a photo-thermosensitive genic male sterile line Peiai64S. Scale bar: 50 μm.

Fig 3b

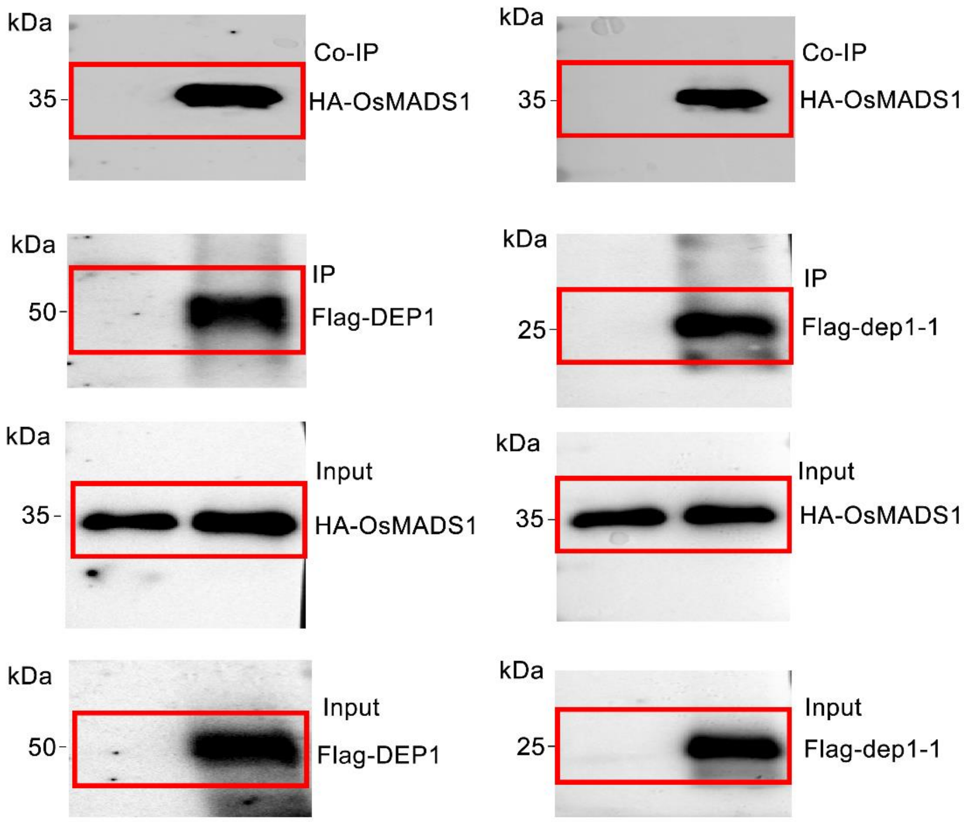


Supplementary Fig 2b

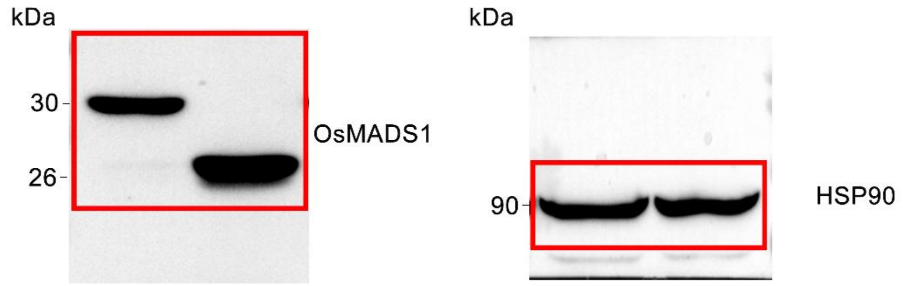


**Supplementary Figure 10. Uncropped images of blots presented in the main paper.** Molecular weight markers are indicated in kDa.
