# Supplementary material for: G-protein βγ subunits determine grain size through interaction with MADS-domain transcription factors in rice
Source: Nat Commun. 2018 Feb 27;9:852. doi: 10.1038/s41467-018-03047-9 (PMC5829230; doi:10.1038/s41467-018-03047-9)
Supplement: Supplementary file 3 — Descriptions of Additional Supplementary Files [file 41467_2018_3047_MOESM3_ESM.pdf]

Descriptions of Additional Supplementary Files

File Name: Supplementary Data 1
Description: Sequence and haplotype analysis of the OsMADS1 gene. The file suggests that an insertion-deletion polymorphism in the splice site of the intron 7/exon 8 junction of *OsMADS1*, resulting in an alternatively spliced protein $OsMADS1^{lgy3}$, is common within *O. nivara* accessions and tropical *japonica* germplasm, but it does not appear to occur within the elite indica and temperate japonica rice varieties.

File Name: Supplementary Data 2
Description: The effects of the *lgy3* allele on the physicochemical characteristics of milled rice. The file shows that WYJ7-*lgy3*-*dep1-1* plants produced much better quality in terms of grain length to width ratio and grain chalkiness.

File Name: Supplementary Data 3
Description: The common target genes oppositely regulated by the *lgy3* and *dep1-1* alleles. The file includes comparison of RNA-seq analysis among NIL plants, which revealed a total of 451 genes, which were co-operatively regulated by the *DEP1-OsMADS1* regulatory module.

File Name: Supplementary Data 4
Description: The primer sequences used for map-based cloning and genotyping assays. The file includes the sequence information of primers used for positional cloning and genotyping.

File Name: Supplementary Data 5
Description: The primer sequences used for transgene constructs. The file includes the detail information of primer sequences used for transgene constructs.

File Name: Supplementary Data 6
Description: The primer sequences used for qRT-PCR and ChIP assays. The file includes the detail information of primers used for quantitative PCR and ChIP assays.
